# ENT/Audiology Department of Samoa welcomes the introduction of the Pneumococcal Conjugate Vaccine (PCV) into the national childhood immunisation program.

**DOI:** 10.7189/jogh.12.02002

**Published:** 2022-05-30

**Authors:** Annette Kaspar, Akshaya Mishra, Amanda Leach, Sione Pifeleti

**Affiliations:** 1 ENT Department, Tupua Tamasese Meaole Hospital, Ministry of Health, Apia, Samoa; 2 Ear Health Research Program, Child Health Division, Menzies School of Health Research, Darwin, Australia; 3 Expanded Program on Immunisation, UNICEF – Samoa Office, Apia, SAMOA

In October 2021, the pneumococcal conjugate vaccine (PCV) was introduced into the national immunisation program of Samoa, a Polynesian nation of the Pacific Islands [1]. The immunisation schedule is three doses of PCV for infants (6, 10, and 14 weeks of age), or two catch-up doses with an eight-week interval for toddlers (12-24 months of age). The decision to implement routine PCV into the Samoan childhood immunisation program was based on the national disease burden of pneumonia, as well as the results of a health economic analysis demonstrating the cost-effectiveness of PCV introduction [1]. Pneumococcal bacteria cause several infectious diseases, especially among infants and toddlers, and the PCV should also reduce the avoidable burden of these conditions (**Figure 1**). A further benefit of the childhood PCV program will be the development of “herd protection” due to reduced transmission of pneumococci from vaccinated infants to older non-vaccinated family members, particularly the elderly (**Figure 1**).

**Figure 1:**
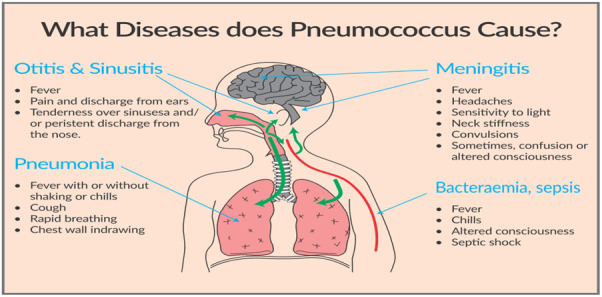
Overview of diseases caused by pneumococcus bacteria. (Source: Dr Akshaya Mishra, Samoa Office, UNICEF)

Samoa has selected the 13-valent PCV which will protect against the 1, 3, 4, 5, 6A, 6B, 7F, 9V, 14, 18C, 19A, 19F, and 23F serotypes of the pneumococcus pathogen. In the above-described schedule, the PCV is recommended for premature neonates once they have reached the recommended chronological age for PCV administration, as well as for immunodeficient children who are the most at risk for developing pneumococcal disease. The 13-valent PCV has a high safety profile, and the only contraindications are previous allergic reactions to any component of the vaccine and/or infants with a moderate or severe illness (temperature over 39°C).

The Ear, Nose and Throat (ENT)/Audiology Department of Samoa welcomes the addition of PCV to the national immunisation program. It is a major public health strategy that should positively impact the rates of ear disease and associated hearing loss among Samoan infants and young children [2]. The Pacific Island region is estimated to have among the highest levels of otitis media (middle ear infections) and secondary hearing loss in the world [3], and a public health approach has already been advocated for its prevention, early identification, and management in the Pacific Islands [4]. We therefore welcome this additional strategy in our battle against childhood chronic otitis media and avoidable hearing loss during the most critical years of speech and language development.

## PCV AND THE PREVENTION OF OTITIS MEDIA AND HEARING LOSS AMONG CHILDREN

The theory behind the PCV and its prevention of otitis media and associated hearing loss is fairly straightforward. Ear disease begins early in Pacific Islander populations, with the first episode of acute otitis media occurring within the first few weeks/months of life following early nasopharyngeal colonisation by pneumococcal pathogens and their migration to the middle ear cavity via the Eustachian tube. The evidence is that the PCV reduces the nasopharyngeal load of pneumococcal pathogens, and therefore reduces the spread to the middle ear and the risk of (acute) otitis media. The causal pathogen *Streptococcus pneumoniae* (*S. pneumoniae*) is classified into 93 known serotypes, and the challenge for vaccine developers is to ensure the recommended PCV targets the relevant *S. pneumoniae* serotypes for the intended population.

Australian Aboriginal children have the highest rate of otitis media disease in the world, and the reality of an “Otitis Media Vaccine” has long been investigated. Studies from Australia found that, while the introduction of the PCV reduced the rate of ear infections for the targeted pneumococcal serotypes, ear disease remained high due to being replaced by previously less common non-vaccine pneumococcal serotypes, and persistence of other pathogens causing middle ear infections (predominantly non-typeable *Haemophilus influenzae*) [5,6]. Researchers note that such replacement has not been considered for invasive pneumococcal disease (IPD). Researchers further recommend that the implementation of the PCV must be coupled with constant monitoring and evaluation of serotypes to ensure maximum efficacy of the immunisation.

### Literature review on PCV in the Pacific Islands

A literature review found seven papers investigating PCV in Pacific Island nations, with 5 from Fiji [7-11], one from Papua New Guinea [12], and another from Tonga [13]. Three of the papers from Fiji evaluated the effect of 10-valent PCV introduction in October 2012 and found that results were overall very positive. The prevalence of vaccine-serotype carriage reduced among infants, toddlers, young children, and caregivers, and the density of PCV10 and non-PCV10-serotypes was significantly lower in PCV10-vaccinated 12-23-month-olds than their non-PCV10-vaccinated counterparts [8]. A time-series analysis also found a dramatic reduction in pneumonia hospital admissions following the introduction of the PCV10 for the 2-59 months age group [9]. The significant factors contributing to pneumococcal carriage and density continue to be young age, residential location, living with young children, low family income, and symptoms of upper respiratory tract infection [10]. This finding highlights the ongoing importance of environmental public health and improved standards of living. In a separate study, pneumococcal nasopharyngeal carriage was higher among newborns/infants born by vaginal delivery in Fiji than those born by Caesarean section, supporting the hypothesis that vertical pneumococcal transmission occurs through exposure to vaginal microbiome [7]. The fifth paper from Fiji was an earlier paper reporting the seven-valent PCV [11].

The study from Papua New Guinea found a limited reduction in the nasopharyngeal carriage of PCV7-type pneumococci among newborns and infants receiving PCV7 compared to those not receiving it [12]. The authors attribute this finding to the early age of dense nasopharyngeal carriage of a wide diversity of non-PCV7 serotypes and a relatively low proportion of PCV7 serotypes in this cohort, resulting in low statistical power. Again, the lesson we can draw from the Papua New Guinean experience is that PCV is but one part of a package of public health care measures: childhood immunisations should not overshadow the role of environmental health measures in reducing the risk of infections.

The study from Tonga aimed to evaluate hospital admissions of invasive pneumococcal disease in a nation that has no pneumococcal vaccine program [13]. The calculated incidence rates were 113/100 000 for children under 2 years, 50/100 000 for children under 5 years, and 25/100 000 for children under 15 years. The case fatality rate for children under 5 years was 25%. The authors urged the introduction of PCV into the national immunisation schedule for children.

### Ear disease, hearing loss, and PCV introduction in Samoa – the way forward

At this time, the foreseeable challenge in Samoa is restoring public confidence in childhood immunisations and achieving optimal immunisation coverage rates through health promotion activities [14]. Although the Samoan national program includes vaccines against the major infections that cause permanent (sensorineural) hearing loss (ie, measles, meningitis), vaccine hesitancy rates remain high since the tragedy that led to the measles epidemic of 2019 [15,16]. The ENT/Audiology Department acknowledges the vital role of health promotion in addressing childhood hearing loss in the Pacific Islands, and we fully support a closer collaboration between our two departments in working towards higher immunisation coverage rates and lower childhood otitis media and hearing loss rates [14].

The most important hearing health awareness event of the year for the Samoan ENT/Audiology Department is World Hearing Day (March 3rd). This annual event represents an opportunity to create awareness of hearing health issues, as well as promote simple public health messages that should reduce the avoidable burden of hearing loss. A key message to caregivers of infants and young children is to keep the nose clean and dry in order to prevent ear infections. In 2022, the ENT/Audiology Department will be able to announce that the PCV is a new national strategy that should positively impact on ear disease and hearing loss, and thus re-iterate the importance of complying with immunisation program recommendations.

It will be difficult to evaluate the direct impact, or any impact at all, of the introduction of PCV in Samoa on the prevalence of ear disease and hearing loss among infants and young children. Such a study would require equipment and human resources that are currently unavailable in the country, and which are unlikely to be available soon due to the COVID-19 pandemic international travel restrictions (ie, baseline and post-PCV introduction of microbiology/serotyping of causal pathogens of ear infections). Furthermore, there is no epidemiological data on ear and hearing health in Samoa. The ENT/Audiology Department aims to address this knowledge gap by conducting a clinical survey of the pattern and prevalence of ear disease among infants, pre-school children, and primary school students in Samoa during 2022-2023. Should a similar study be repeated in 5 years’ time to monitor for any changes, a reduction in ear disease rates will be difficult to attribute directly to the introduction of the PCV.

However, our discussion should not lose sight of the bigger picture. The implementation of PCV will reduce pneumococcal diseases and their morbidity and mortality rates. Samoan children will be healthier as a result, and health promotion messaging should continue to focus on immunisation confidence, healthy nutrition/lifestyles, and environmental health and hygiene. From the ENT/Audiology perspective, the anticipated reduction of upper respiratory tract infections should reduce secondary ear diseases and hearing loss.

## CONCLUSIONS

The ENT/Audiology Department of Samoa welcomes the introduction of the 13-valent PCV into our national immunisation program. It is an important strategy for reducing middle ear infections and avoidable hearing loss among infants and young children, and we encourage our Pacific Island neighbours to similarly embrace the PCV where possible.
